# The gene expression of numerous SLC transporters is altered in the immortalized hypothalamic cell line N25/2 following amino acid starvation

**DOI:** 10.1002/2211-5463.12181

**Published:** 2017-01-11

**Authors:** Sofie V. Hellsten, Emilia Lekholm, Tauseef Ahmad, Robert Fredriksson

**Affiliations:** ^1^ Department of Pharmaceutical Bioscience, Molecular NeuropharmacologyUppsala UniversitySweden; ^2^ Department of Neuroscience, Functional PharmacologyUppsala UniversitySweden

**Keywords:** amino acid starvation, amino acid transporter, gene expression, solute carriers

## Abstract

Amino acids are known to play a key role in gene expression regulation, and in mammalian cells, amino acid signaling is mainly mediated via two pathways, the mammalian target of rapamycin complex 1 (mTORC1) pathway and the amino acid responsive (AAR) pathway. It is vital for cells to have a system to sense amino acid levels, in order to control protein and amino acid synthesis and catabolism. Amino acid transporters are crucial in these pathways, due to both their sensing and transport functions. In this large‐scale study, an immortalized mouse hypothalamic cell line (N25/2) was used to study the gene expression changes following 1, 2, 3, 5 or 16 h of amino acid starvation. We focused on genes encoding solute carriers (SLCs) and putative SLCs, more specifically on amino acid transporters. The microarray contained 28 270 genes and 86.2% of the genes were expressed in the cell line. At 5 h of starvation, 1001 genes were upregulated and 848 genes were downregulated, and among these, 47 genes from the SLC superfamily or atypical SLCs were found. Of these, 15 were genes encoding amino acid transporters and 32 were genes encoding other SLCs or atypical SLCs. Increased expression was detected for genes encoding amino acid transporters from system A, ASC, L, N, T, xc‐, and y+. Using GO annotations, genes involved in amino acid transport and amino acid transmembrane transporter activity were found to be most upregulated at 3 h and 5 h of starvation.

**Abbreviations**
AAR: amino acid responsive
AARE: amino acid responsive element
AS: asparagine synthetase
ATF: activating transcription factor
CHOP: CCAAT/enhancer‐binding protein homologous protein
eiF2α: eukaryotic initiation factor 2α
GCN2: general control nonderepressible 2
MFS: major facilitator superfamily
mTORC1: mammalian target of rapamycin complex 1
NSRE: nutrient‐sensing responsive element
SLC: solute carrier

It is vital for cells to have system for sensing amino acid levels in order to regulate protein and amino acid synthesis and catabolism 1. In mammalian cells, amino acid signaling is, in principal, mediated via two pathways and the amino acid availability plays a key role in the regulation of gene expression 2. The mammalian/mechanistic target of rapamycin complex 1 (mTORC1) pathway and the amino acid responsive (AAR) pathway can control the protein synthesis by either upregulate or downregulate it, depending on the levels of amino acids 3. The mTORC1 pathway is activated when the cell has sufficient amino acid levels and function as a sensor for adequate amino acid concentrations, in order to maintain protein synthesis and cellular growth 4. The AAR pathway, on the other hand, is activated when the cell has limited access to amino acids, which results in inhibition of general protein synthesis 5. When the AAR pathway is activated, the general control nonderepressible 2 (GCN2) kinases are activated by binding to uncharged tRNA, which accumulate during deprivation 3, 6. In turn, these kinases inactivate the eukaryotic initiation factor 2α (eiF2α) by phosphorylation resulting in inhibition of protein translation 7. The activation transcription factor 4 (ATF4), and to some extent ATF2, are transcriptionally upregulated by the inhibition of protein synthesis and these factors play key roles in the regulation of gene expression 8, 9. These transcription factors bind elements, termed amino acid responsive element (AARE), nutrient‐sensing element 1 (NSRE1) or NSRE2, which are short sequences of nucleotides, and genes holding these elements are upregulated 5, 6, 10, 11. The first responsive elements identified were found in the genes encoding CCAAT/enhancer‐binding protein homologous protein (CHOP) and asparagine synthetase (AS) 11, 12, 13, which are induced during amino acid deprivation 14, 15. The solute carrier (SLC) superfamily is the largest family of transport proteins in mammals, with 456 members 16 divided into 52 families in human 17. Out of these SLCs, over 60 have been found to transport amino acids, and in addition, another 40 orphans are closely related to known amino acid transporters, suggesting there could be over 100 amino acid transporters in human 18. The SLCs are ATP‐independent uniporters, symporters, or antiporters, and further divided into different transport systems (e.g. system A, L, N, and xc‐) depending on transport mechanism and substrate profile 19. Transporters are thought to be important regulators in nutrient sensing and signaling 20 and amino acid transporters have been suggested to function as transceptors, transporters with both transport and receptor functions 21. They have the capacity to regulate the intracellular amino acid concentrations, and in addition, also sense alterations in extracellular amino acid levels 21. Genes encoding amino acid transporters, with characterized responsive elements, from several SLC families, have previously been found to be induced upon amino acid starvation, e.g. *Slc7a1* 22, *Slc7a5* 23, *Slc7a11* 24, *Slc1a4* 25, 26, *Slc1a5* 26, *Slc3a2* 26, and *Slc38a2* 27. The regulation has been studied in different cells, deprived of one or several amino acids, e.g. in mouse NIH3T3 cells, the system xc‐ activity and *Slc7a11* mRNA were increased 24, in rat hepatic WB cells the *Slc7a5/Slc3a2* expression and activity was induced 23, in rat C6 glioma cells *Slc7a1* was upregulated 28 and *Slc38a2* was found to be induced in both human HepG2 hepatoma cells 29 and human trophoblast BeWo cells 30. However, how SLC encoding genes respond to amino acid starvation has not previously been studied on a larger scale.

In this study, the immortalized mouse embryonic hypothalamic cell line N25/2 was deprived of all amino acids for 1, 2, 3, 5, or 16 h. Hypothalamus has a well‐established role in sensing amino acid levels 31, 32 and therefore we chose to deprive a hypothalamic cell line of amino acids. The aim was to, on a large scale, study the regulation of genes encoding amino acid transporters and putative amino acid transporters from the SLC superfamily or atypical SLCs, using microarray analysis.

## Materials and methods

### Culturing of the immortalized hypothalamic cell line N25/2

The immortalized mouse embryonic hypothalamic cell line, N25/2, (mHypoE‐N25/2, CEDARLANE, Burlington, ON, Canada) was cultured in Dulbecco's modified Eagle's medium (DMEM) (Gibco^®^, Life technologies, Carlsbad, CA, USA) supplemented with 10% fetal bovine serum (FBS), (Gibco^®^, Life technologies), 1% penicillin‐streptomycin, liquid (Gibco^®^, Life technologies), and 1% Fungizone^®^ Antimycotic (Amphotericin B) (Gibco^®^, Life technologies) at 37 °C in a humidified atmosphere of 5% CO_2_, 95% air. Cells were grown to 70–90% confluence in Nunclon surface dishes 150 × 20 mm (Thermo Scientific, Waltham, MA, USA).

### Amino acid deprivation of the immortalized hypothalamic cell line N25/2

Medium for the experiment was prepared with Earle's balanced salt solution (EBSS) (Gibco^®^, Life technologies), 1 mm sodium pyruvate 100 mm (Gibco^®^, Life technologies), 4X MEM vitamin solution (100X) liquid (Gibco^®^, Life technologies). Neither the control medium nor the starved medium was supplemented with FBS. Following amino acids were added to the EBSS medium containing amino acids, 0.4 mm glycine, 0.4 mm l‐arginine, 0.2 mm l‐cystine, 4.0 mm l‐glutamine, 0.2 mm l‐histidine, 0.8 mm l‐isoleucine, 0.8 mm l‐leucine, 0.8 mm l‐lysine, 0.2 mm l‐methionine, 0.4 mm l‐phenylalanine, 0.4 mm l‐serine, 0.8 mm l‐threonine, 0.08 mm l‐tryptophan, 0.4 mm l‐tyrosine, and 0.8 mm l‐valine (Sigma‐Aldrich, St. Louis, MO, USA), the same amino acid concentrations as in the commercially available DMEM medium. The complete DMEM medium was removed and replaced with EBSS medium lacking amino acids or EBSS medium supplemented with amino acids. The cells were treated in the different media for 1 h (*n* = 1), 2 h (*n* = 1), 3 h (*n* = 1), 5 h (*n* = 4), or 16 h (*n* = 1) before RNA was extracted with RNeasy Midi Kit (Qiagen, Hilden, Germany), following the manufacture's protocol.

### Microarray analysis of gene expression

The RNA concentration was measured with ND‐1000 spectrophotometer (NanoDrop Technologies, Wilmington, DE, USA) and RNA quality was evaluated using the Agilent 2100 Bioanalyzer system (Agilent Technologies Inc, Palo Alto, CA, USA). 250 ng of total RNA from each sample was used to produce amplified and biotinylated sense‐strand cDNA from the entire expressed genome according to the Ambion WT Expression Kit (P/N 4425209 Rev C 09/2009) and Affymetrix GeneChip^®^ WT Terminal Labeling and Hybridization User Manual (P/N 702808 Rev. 3, Affymetrix Inc., Santa Clara, CA, USA). GeneChip^®^ ST Arrays (GeneChip^®^ Mouse Gene 1.0 ST Array) were hybridized for 16 h in a 45 °C incubator, and rotated at 60 rpm. According to the GeneChip^®^ Expression Wash, Stain and Scan Manual (PN 702731 Rev 3, Affymetrix Inc.), the arrays were then washed and stained using the Fluidics Station 450 and finally scanned using the GeneChip^®^ Scanner 3000 7G. Analysis of the gene expression data was carried out in the freely available statistical computing language ʀ (http://www.r-project.org) using packages available from the Bioconductor project (www.bioconductor.org). The raw data was normalized using the robust multi‐array average (RMA) method first suggested by Li and Wong in 2001 33, 34. In order to search for the differentially expressed genes between the *X* samples and the *Y* samples group an empirical Bayes moderated *t*‐test was then applied 35, using the ‘limma’ package 36. To address the problem with multiple testing, the *P*‐values were adjusted using the method of Benjamini and Hochberg 37. The quadruplicates at 5 h of starvation (*n* = 4); singlets in each treatment group was run at a time followed by microarray analysis, while 5‐h triplicates together with singlets from the other incubation times (1, 2, 3, and 16 h) were run and analyzed with microarray at a different time. The microarray data from 5 h were combined and analyzed together as one set of data of quadruplicates. The array was performed at the Array and Analysis Facility, Science for Life Laboratory at Uppsala Biomedical Center (BMC), Husargatan 3, 751 23 Uppsala, Sweden. The microarray data can be found in the NCBI‐GEO database with accession number GSE61402.

### Microarray data analysis

Following programs/websites were used to analyze the data. ease version 2.0 was used to analyze the data across GO annotations. blast p version 2.2.3 and the webpage http://www.ensembl.org were used to find all SLCs and atypical SLCs in the dataset.

### Heat map analysis

genesis version 1.7.6 was used to create a heat map over the gene expression alterations (1–16 h) for all 47 genes encoding SLCs or atypical SLCs, found to be significantly altered at 5 h of starvation. The log 2 fold change value for 5 h, and the difference between the log 2 values of expression in controls and starved cells for 1, 2, 3, and 16 h were used. Two of the genes (*Slc3a1*and *Slc38a1*) had two probes on the GeneChip with significant gene expression changes, and the probe with the lowest adjusted *P*‐value was selected for the expression analysis.

### qPCR analysis of gene expression

The RNA concentration was determined using a NanoDrop ND‐1000 Spectrophotometer (Thermo Fischer Scientific) and cDNA was synthesized using the SuperScript^®^ III Reverse Transcriptase Kit (Invitrogen, Waltham, MA, USA) following the manufacture's protocol before diluted to a concentration of 5 ng·μL^−1^. The cDNA samples were analyzed using qPCR on MyiQ thermal cycler (Bio‐Rad Laboratories, Hercules, CA, USA). All primers were designed with beacon designer v.8 (Premier Biosoft, Palo Alto, CA, USA), and sequences can be found in Table 1. Housekeeping genes used for normalization were mouse *mβ‐Actin*,* mβ‐Tubulin*, and *mGlycerylaldehyde 3‐phosphate dehydrogenase*. The qPCR reactions for all primer pairs except *Slc38a7* and *Slc23a3* were run in a total volume of 12.5 μL with 5 ng cDNA using BR SYBR^®^ Green SuperMix for IQ™ Systems (Quanta Biosciences, Gaitherburg, MD, USA) because of requirement of cDNA amplification. For *Slc38a7* and *Slc23a3*, SYBR^®^ Select Master Mix kit (Applied Biosystems^®^, Waltham, MA, USA) was used for qPCR reaction in a total volume of 20 μL with 5 ng of cDNA. The amplification was performed under following conditions; for primers with annealing temperature ≥60 °C; initial denaturation 95 °C for 2 min followed by 40 cycles of: denaturation at 95 °C for 15 s, annealing/elongation at 60 °C for 1 min. For primers with annealing temperature ≤60 °C; initial denaturation 95 °C for 2 min, followed by 40 cycles of: denaturation at 95°C for 15 s, annealing at 55–60 °C for 15 s, and elongation at 72 °C for 1 min. In both cases, the cycling was followed by melt curve performance starting at 55 to 95 °C with steps of 0.5 °C. The experiment was performed in triplicates. Water was used as a negative control and cDNA from a whole mouse brain was included on each plate as a positive control. For *Slc16a2*,* Slc40a1*, and *Mfsd2a*, samples from 16‐h starvation were analyzed using 40 ng cDNA per qPCR reaction combined with 0.05 μL of each primer (100 pmol·μL^−1^), 2 μL 10X DreamTaq buffer (Thermo Fischer Scientific), 0.2 μL of 25 mm dNTP mix (Thermo Fischer Scientific), 1 μL DMSO, 0.5 μL SYBR Green (Invitrogen), and 0.08 μL of Dream Taq (5U·μL^−1^, Thermo Fisher scientific). The volume was adjusted to 20 μL with sterile water. qPCR was run using initial denaturation for 30 s at 95 °C, 50 cycles of 10 s at 95 °C, 30 s at 52–55 °C (optimal temperature depending on primer), and 30 s at 72 °C. A melting curve was performed starting at 55 °C for 81 cycles at 10‐s interval and a temperature increase of 0.5 °C per cycle. All q‐PCR were run in quadruplicates and a negative control was included on each plate.

**Table 1:** Primers used for the qPCR reactions

Primer	Forward/Reverse
*Slc7a11* (NM_011990)	tgg aac tgc tcg taa tac/gtt cag gaa ttt cac att ga
*Slc40a1* (NM_016917)	ctt tgc tgt tgt tgt ttg/gag agg aac cga aga tag
*Mfsd11* (NM_178620)	cta tgt ttg tca gtg gtt tg/aga tgc tgt gta gaa gga
*Slc25a36* (NM_138756)	acc tgt gcc aca acc ata/atc cat agc ctt ctt ctt gaac
*Slc6a9* (NM_008135)	ttt ccc ata cct ctg cta/aaa gct cca tga aga aga
*Slc7a1* (NM_007513)	aat tat cat ctt aac agg actg/gac cag gac att gat aca
*Slc23a3* (NM_194333)	tct tca act tca act cac at/aca aag gca gag atg aac
*Slc9a9* (NM_177909)	tga tat tga tag tgg aac tgtct/ctt ggt cgg tga tgt tga
*Slc25a33* (NM_027460)	agt tcc tct ggc ttc tttg/tcc tga tga cct cgt gtg
*Slc38a7* (NM_172758)	tag cca ttg cgg tct atac/gct cct tcg aca tca cag
*Slc16a9* (NM_025807)	ccc aat atc tac ttt ctg ttt/cgt cgc tgt gta taa tag
*Slc16a2* (NM_009197)	ttt ccc ttc ctc atc aaa/gta agt gag tga gag cag
*Mfsd2a* (NM_029662)	cta tgt caa gct cat tgc/gaa gtc caa ggt ata ggt
*Slc43a2* (NM_173388)	gtt tat gca cag tgt gtt/aag atg gag gta tag agg
*mβ‐Actin (mActb)*	cct tct tgg gta tgg aat cct gtg/cag cac tgt gtt ggc ata gag g
*mβ‐Tubulin (mbTUB)*	agt gct cct ctt cta cag/tat ctc cgt ggt aag tgc
*mGlycerylaldehyde 3‐phosphate dehydrogenase (mGAPDH)*	gcc ttc cgt gtt cct acc/gcc tgc ttc acc acc ttc

### qPCR data analysis and relative expression calculations

The MyIQ software (Bio‐Rad Laboratories) was used to obtain the qPCR threshold cycle Ct‐values and melt curve data. The melting curves were compared to the positive and negative control to verify that only one product was amplified. The triplicates for the raw Ct‐values were compared and outliers were excluded if the difference was greater than 0.99 between the Ct triplicates. The efficiency for each primer pair was determined using LinRegPCR v7.5. The average qPCR primer efficiency and standard deviation for each primer was calculated after outliers were removed using Grubbs test (GraphPad Software, San Diego, CA, USA). The delta Ct‐method was used to transform the mean of raw Ct‐values into relative quantities with standard deviations. Geometric means of all three housekeeping genes were calculated and used for normalization. Unpaired *t*‐tests (*≤0.05, **≤0.01, ***≤0.001) were performed using GraphPad Prism 5 between the control cells and the starved cells.

## Results

### Amino acid starvation of the immortalized hypothalamic cell line N25/2

The hypothalamic cell line N25/2 was starved of all amino acids for 1, 2, 3, 5, and 16 h. Shorter times were chosen because this would possibly enable detection of changes in expression of genes involved in an earlier response. A starvation time of 16 h was chosen based on previous studies in other cell lines 25, 38, where similar times resulted in marked expression level alterations of amino acid transporters. The microarray GeneChip had 28 270 probes and 86.2% of the genes had detectable expression in the cell line (i.e. value of expression >5). About 1849 genes were significantly (adj. *P*‐value <0.01) up‐ or downregulated at 5 h of amino acid starvation compared with controls, and of these, 1001 genes were upregulated and 848 genes were downregulated. Among these, 47 transcripts encoding SLCs or atypical SLCs were found. We provide expression levels for all genes on the array and the data can be found in the NCBI‐GEO database with accession number GSE61402.

### Principal component analysis

**Figure 1:**
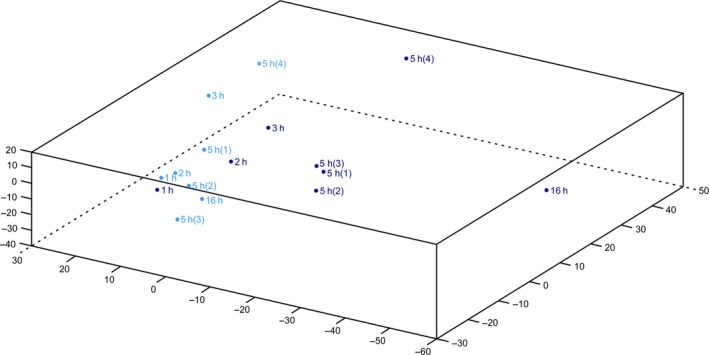
Principal component analysis of the expression levels of all genes following amino acid starvation at 1, 2, 3, 5, or 16 h in the hypothalamic cell line N25/2. The control cells, treated with amino acids (in light blue) clusters to the left, while the amino acid starved cells (in dark blue) shift to the right in the figure, with more shift with increased time of amino acid deprivation.

The gene expression for the entire array, at all times, was analyzed in a principal component analysis (PCA) plot (Fig. 1). The nonstarved cells cluster to the left in the figure, while the amino acid deprived cells shift to the right, with more shift with increased time of deprivation.

### Type of SLC transporters and putative transporters

**Figure 2:**
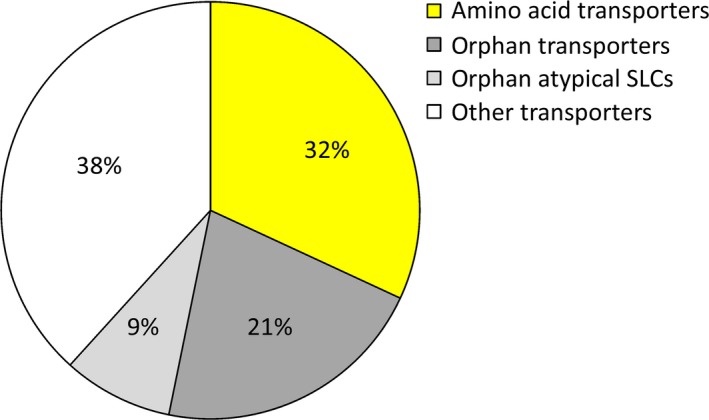
A pie chart of the SLC transporters found to have altered gene expression. The pie chart displays that 32% of the genes encode amino acid transporters, while 38% of the genes encode other nonamino acid‐transporting SLCs, e.g., transporters for thiamine, iron, sugars, vitamins, ions, fatty acids, UTP, pyrimidine nucleotides, and hormones. Thirty percent of the transporters are orphans, and among these 21% are orphan SLCs and 9% are atypical SLCs.

The 47 SLC or atypical SLC genes found, were divided into four groups based on what type of transporters they encode (Fig. 2). Among these, 15 genes encoded amino acid transporters from the SLC family, 10 genes encoded orphan SLCs, 4 genes encoded atypical orphan SLCs, and 18 genes encoded nonamino acid SLC transporters, e.g. transporters for thiamine, iron, sugar, vitamin, ion, fatty acid, UTP, pyrimidine nucleotide, and hormone. In Table 2, a summary of the amino acid transporter encoding genes are presented. In Table 3, the genes encoding orphan SLCs, atypical orphan SLCs and nonamino acid SLC transporters are listed.

**Table 2:** Up‐ and downregulated amino acid transporter encoding genes belonging to the SLCs. Data from 5 h of amino acid starvation. The information about substrate/system is from SLC tables, http://slc.bioparadigms.org/

Clan	Pfam family	Gene	Substrate/System	Adj.*P*‐value	log2Fold Change	Probe ID
Amino acid‐Polyamine‐organoCation (APC) superfamily	AA_permease_2	*Slc7a11*	cystine (anionic form), l‐glutamate, system xc‐	2.4E‐06	3.2	10498024
	AA_permease					
APC superfamily	SNF	*Slc6a9*	glycine	2.3E‐05	1.8	10507500
APC superfamily	AA_permease_2	*Slc7a1*	cationic l‐amino acids, system y+	4.1E‐05	1.7	10535852
	AA_permease					
	AA_permease_C					
–	SDF	*Slc1a4*	l‐Ala, l‐Ser, l‐Cys, l‐Thr, system ASC	5.6E‐07	1.2	10384539
APC superfamily	AA_permease_2	*Slc7a5*	large neutral l‐amino acids, T_3_, T_4_, l‐DOPA, BCH, system L	5.1E‐05	1.1	10582275
	AA_permease					
–	SDF	*Slc1a5*	l‐Ala, l‐Ser, l‐Cys, l‐Thr, l‐Gln, l‐Asn, system ASC	7.5E‐05	1.1	10550332
APC superfamily	Aa_trans	*Slc38a7*	Gln, His, Ser, Ala, Asn	2.2E‐05	1.0	10580957
	Trp_Tyr_perm					
APC superfamily	Aa_trans	*Slc38a1*	Gln, Ala, Asn, Cys, His, Ser, system A	9.5E‐05	0.9	10431872
Tim Barrel Glycosyl hydrolase superfamily	Alpha‐amylase	*Slc3a2*	system L, y+L, xc‐ and ASC with light subunits SLC7A5‐8 and SLC7A10‐11	9.9E‐06	0.8	10465772
APC superfamily	Aa_trans	*Slc38a2*	Ala, Asn, Cys, Gln, Gly, His, Met, Pro, Ser, system A	1.2E‐05	0.6	10431894
–	Mito_carr	*Slc25a26*	S‐adenosyl‐methionine, S‐adenosyl‐homocysteine	3.2E‐04	0.5	10540215
Major facilitator superfamily (MFS)	PTR2	*Slc15a4*	His, di‐ and tri‐peptides, protons	4.2E‐03	0.3	10533993
	MFS_1					
MFS	MFS_1	*Slc16a10*	aromatic amino acids, T_3_, T_4_	9.8E‐03	0.3	10368720
Tim Barrel Glycosyl hydrolase superfamily	Alpha‐amylase	*Slc3a1*	system b0,+,Heterodimerizes with light subunit SLC7A9, cationic amino acids, large neutral amino acids,	1.9E‐05	−0.6	10453373
MFS	MFS_1	*Slc43a2*	l‐BCAAs, amino alcohols	7.3E‐03	−0.8	10378649

**Table 3:** Up‐ and downregulated genes encoding nonamino acid‐transporting SLCs, orphan SLCs, or atypical SLCs. Data from 5 h of amino acid starvation. The information about substrate/system is from SLC tables, http://slc.bioparadigms.org/

Clan	Pfam family	Gene	Substrate	Adj.*P*‐value	log2Fold Change	Probe ID
MFS	FPN1	*Slc40a1*	ferrous iron	2.1E‐03	1.6	10354374
APC superfamily	Xan_ur_permease	*Slc23a3*	O	2.6E‐04	1.5	10355717
MFS	Sugar_tr	*Slc2a12*	glucose	1.8E‐06	1.2	10368229
	MFS_1					
–	Mito_carr	*Slc25a33*	UTP	2.8E‐05	1.2	10518726
CPA/AT transpoter family	Na_H_exchanger	*Slc9a9*	Na^+^, K^+^, H^+^	1.5E‐05	1.0	10587854
Drug/metabolite transporter superfamily (DMT)	Cation_efflux	*Slc30a1*	O	3.0E‐05	1.0	10352777
MFS	MFS_1	*Mfsd11*	O	3.5E‐05	0.7	10382852
MFS	MFS_1	*Slc16a14*	O	2.3E‐04	0.7	10356240
MFS	Folate_carrier	*Slc19a2*	thiamine	5.7E‐04	0.7	10351259
–	Mito_carr	*Slc25a37*	Fe^2+^	1.4E‐04	0.6	10421172
MFS	MFS_1	*Mfsd7b*	O	3.6E‐04	0.5	10361065
–	Mito_carr	*Slc25a36*	pyrimidine nucleotides	1.2E‐03	0.5	10479979
DMT	Cation_efflux	*Slc30a4*	O	2.3E‐04	0.4	10487021
MFS	MFS_1	*Slc17a5*	sialic acid, other acidic sugars	1.5E‐03	0.4	10595189
–	Mito_carr	*Slc25a30*	O	8.9E‐04	0.4	10421648
APC superfamily/STAS domain superfamily	Sulfate_transp	*Slc26a11*	Cl‐, HCO_3_^−^, SO_4_^2−^, oxalate	6.3E‐03	0.4	10383133
	Sulfate_tra_GLY					
	STAS					
MFS	Folate_carrier	*Slc19a3*	thiamine	3.9E‐03	0.3	10356145
	MFS_1					
MFS	MFS_1	*Mfsd1*	O	2.9E‐03	0.3	10492499
DMT	TPT	*Slc35e1*	O	9.9E‐04	−0.3	10579724
DMT	UAA	*Slc35b1*	O	3.8E‐03	−0.3	10380524
	EamA					
	TPT					
APC superfamily/STAS domain superfamily	Sulfate_transp	*Slc26a2*	SO_4_^2−^, oxalate, Cl^−^	7.9E‐03	−0.3	10459183
	Sulfate_tra_GLY					
	STAS					
–	Mito_carr	*Slc25a38*	O	6.4E‐04	−0.4	10590245
ANL superfamily	AMP‐binding	*Slc27a4*	LCFA, VLCFA	1.4E‐03	−0.4	10470751
APC superfamily/Phosphotransferase/anion transport protein superfamily	HCO3_cotransp	*Slc4a3*	Cl^−^, HCO_3_^−^	7.1E‐03	−0.4	10347697
	Band_3_cyto					
MFS	MFS_1	*Slc16a2*	T2, rT3, T3, T4	2.5E‐04	−0.5	10606186
–	Mito_carr	*Slc25a1*	citrate, isocitrate, malate, PEP	4.0E‐04	−0.5	10438262
DMT	Zip	*Slc39a10*	Zn	3.9E‐05	−0.6	10354389
MFS	Sugar_tr	*Slc2a1*	glucose, galactose, mannose, glucosamine	3.0E‐04	−0.7	10507594
	MFS_1					
–	Mito_carr	*Slc25a10*	malate, phosphate, succinate, sulfate, thiosulfate	2.0E‐05	−0.7	10383395
–	Mito_carr	*Slc25a35*	O	3.1E‐03	−0.8	10377372
MFS	MFS_1	*Mfsd2a*	O	4.1E‐04	−0.9	10516064
	MFS_2					
MFS	MFS_1	*Slc16a9*	O	9.2E‐06	−0.9	10363860

### GO annotations to cluster gene categories

The genes were analyzed using Gene Ontology (GO) annotations for biological process, Table 4, and molecular function, Table 5, using EASE version 2.0. An EASE score ≤0.01 was considered significant, and a maximum of 10 categories for each time were considered. This analysis was performed to pinpoint when the gene clusters related to amino acid transport were regulated. About 1849 genes from each starvation time were used in the GO analysis. The genes from the incubation times 1, 2, 3, and 16 h, with singlets in each treatment group, were sorted by the absolute value of the difference in log 2 expression value between the control and starved cells, and the top 1849 genes were extracted.

**Table 4:** Up‐ and downregulated gene categories in response to amino acid starvation across GO biological processes. For each GO term, the number of genes up‐ or downregulated in response to amino acid starvation is presented

Time (h)	Ontology ID biological process	Gene category	Upregulated	Downregulated
1	GO:0007154	Cell communication	91	
1	GO:0008152	Metabolic process		224
1	GO:0006139	Nucleobase‐containing compound metabolic process		101
1	GO:0016070	RNA metabolism		22
2	GO:0051726	Regulation of cell cycle	23	19
2	GO:0007049	Cell cycle	31	
2	GO:0035556	Intracellular signal transduction	35	
2	GO:0007264	Small GTPase‐mediated signal transduction	15	
2	GO:0006357	Regulation of transcription from RNA polymerase II promoter	15	
2	GO:0009894	Regulation of catabolic process	5	
2	GO:0008283	Cell proliferation	35	35
2	GO:0019222	Regulation of metabolic process	8	
2	GO:0006366	Transcription from RNA polymerase II promoter	17	
2	GO:0000278	Mitotic cell cycle	15	
2	GO:0006355	Regulation of transcription, DNA‐dependent		60
2	GO:0006351	Transcription, DNA‐dependent		62
2	GO:0050794	Regulation of cellular process		15
2	GO:0050789	Regulation of biological process		15
2	GO:0009653	Anatomical structure morphogenesis		41
2	GO:0007266	Rho protein signal transduction		5
3	GO:0046942	Carboxylic acid transport	7	
3	GO:0015849	Organic acid transport	7	
3	GO:0006865	Amino acid transport	6	
3	GO:0015837	Amine transport	6	
3	GO:0006355	Regulation of transcription, DNA‐dependent	49	
3	GO:0051726	Regulation of cell cycle	16	
3	GO:0008152	Metabolic process		221
3	GO:0008283	Cell proliferation		43
3	GO:0007049	Cell cycle		32
3	GO:0009101	Glycoprotein biosynthetic process		9
3	GO:0009058	Biosynthetic process		47
5	GO:0008152	Metabolic process	231	
5	GO:0006412	Translation	20	
5	GO:0009058	Biosynthetic process	57	53
5	GO:0006139	Nucleobase‐containing compound metabolic process	103	
5	GO:0009451	RNA modification	12	
5	GO:0015849	Organic acid transport	9	
5	GO:0046942	Carboxylic acid transport	9	
5	GO:0009059	Macromolecule biosynthetic process	45	
5	GO:0006396	RNA processing	22	
5	GO:0006413	Translational initiation	8	
5	GO:0016125	Sterol metabolic process		9
5	GO:0008203	Cholesterol metabolic process		8
5	GO:0016126	Sterol biosynthetic process		6
5	GO:0030036	Actin cytoskeleton organization		9
5	GO:0006996	Organelle organization		25
5	GO:0006066	Alcohol metabolic process		16
5	GO:0030029	Actin filament‐based process		9
5	GO:0009101	Glycoprotein biosynthetic process		10
5	GO:0007010	Cytoskeleton organization		21
16	GO:0006139	Nucleobase‐containing compound metabolic process	106	
16	GO:0006396	RNA processing	28	
16	GO:0016070	RNA metabolic process	28	
16	GO:0008152	Metabolic process	195	
16	GO:0009451	RNA modification	13	
16	GO:0006399	tRNA metabolic process	13	
16	GO:0006520	Cellular amino acid metabolic process	17	
16	GO:0007049	Cell cycle	31	
16	GO:0006400	tRNA modification	10	
16	GO:0009308	Amine metabolic process	19	
16	GO:0006695	Cholesterol biosynthetic process		9
16	GO:0007275	Multicellular organismal development		108
16	GO:0009887	Organ morphogenesis		70
16	GO:0008203	Cholesterol metabolic process		12
16	GO:0016126	Sterol biosynthetic process		9
16	GO:0009653	Anatomical structure morphogenesis		73
16	GO:0007155	Cell adhesion		44
16	GO:0016125	Sterol metabolic process		12
16	GO:0006629	Lipid metabolic process		39
16	GO:0008610	Lipid biosynthetic process		21

**Table 5:** Up‐ and downregulated gene categories in response to amino acid starvation across GO molecular function. For each GO term, the number of genes up‐ or downregulated in response to amino acid starvation is presented

Time (h)	Ontology ID Molecular function	Gene category	Upregulated	Downregulated
1	GO:0003676	Nucleic acid binding		100
2	GO:0005515	Protein binding	73	
2	GO:0005083	Small GTPase regulatory/interacting protein activity	12	
2	GO:0030234	Enzyme regulator activity	25	
2	GO:0003924	GTPase activity	14	
2	GO:0030695	GTPase regulator activity	12	
2	GO:0003677	DNA binding	58	
2	GO:0005100	Rho GTPase activator activity	3	
2	GO:0003676	Nucleic acid binding		90
2	GO:0003677	DNA binding		66
2	GO:0042379	Chemokine receptor binding		7
2	GO:0008009	Chemokine activity		7
2	GO:0005488	Binding		203
2	GO:0042056	Chemoattractant activity		7
2	GO:0001664	G‐protein‐coupled receptor binding		7
2	GO:0005125	Cytokine activity		16
2	GO:0016757	Transferase activity, transferring glycosyl groups		13
2	GO:0003700	Sequence‐specific DNA binding transcription factor activity		36
3	GO:0003700	Sequence‐specific DNA binding transcription factor activity	36	
3	GO:0015171	Amino acid transmembrane transporter activity	7	
3	GO:0030528	Transcription regulator activity	41	
3	GO:0005275	Amine transmembrane transporter activity	7	
3	GO:0005342	Organic acid transmembrane transporter activity	7	
3	GO:0046943	Carboxylic acid transmembrane transporter activity	7	
3	GO:0003677	DNA binding	54	
3	GO:0016757	Transferase activity, transferring glycosyl groups		18
3	GO:0003676	Nucleic acid binding		101
3	GO:0016740	Transferase activity		62
3	GO:0016758	Transferase activity, transferring hexosyl groups		11
5	GO:0015171	Amino acid transmembrane transporter activity	9	
5	GO:0005275	Amine transmembrane transporter activity	9	
5	GO:0046943	Carboxylic acid transmembrane transporter activity	9	
5	GO:0005342	Organic acid transmembrane transporter activity	9	
5	GO:0005488	Binding	229	
5	GO:0003712	Transcription cofactor activity	14	
5	GO:0008134	Transcription factor binding	15	
5	GO:0003824	Catalytic activity	162	
5	GO:0003676	Nucleic acid binding	94	
5	GO:0045182	Translation regulator activity	10	
5	GO:0005515	Protein binding		98
5	GO:0003779	Actin binding		19
5	GO:0008092	Cytoskeletal protein binding		23
5	GO:0016757	Transferase activity, transferring glycosyl groups		18
5	GO:0008138	Protein tyrosine/serine/threonine phosphatase activity		6
5	GO:0016301	Kinase activity		38
16	GO:0003676	Nucleic acid binding	96	
16	GO:0008168	Methyltransferase activity	12	
16	GO:0016741	Transferase activity, transferring one‐carbon groups	12	
16	GO:0015171	Amino acid transmembrane transporter activity	8	
16	GO:0005275	Amine transmembrane transporter activity	8	
16	GO:0046943	Carboxylic acid transmembrane transporter activity	8	
16	GO:0005342	Organic acid transmembrane transporter activity	8	
16	GO:0008757	S‐adenosylmethionine‐dependent methyltransferase activity	8	
16	GO:0015203	Polyamine transmembrane transporter activity	6	
16	GO:0015175	Neutral amino acid transmembrane transporter activity	4	
16	GO:0008092	Cytoskeletal protein binding		29
16	GO:0003779	Actin binding		23
16	GO:0005509	Calcium ion binding		47
16	GO:0046872	Metal ion binding		72
16	GO:0016491	Oxidoreductase activity		50
16	GO:0008083	Growth factor activity		19
16	GO:0008289	Lipid binding		17
16	GO:0005543	Phospholipid binding		9
16	GO:0005544	Calcium‐dependent phospholipid binding		6
16	GO:0005125	Cytokine activity		22

After 1 h, only one gene category belonging to biological process, ‘cell communication’, was upregulated and many genes involved in metabolic processes were downregulated (Table 4). At this time, only one category of molecular function was downregulated, ‘nucleic acid binding’ (Table 5).

At 2 h of amino acid starvation, genes from the groups ‘regulation of cell cycle’ and ‘cell proliferation’ were found to be up‐ and downregulated in approximately equal proportions. Two of the largest groups of upregulated genes belong to ‘intracellular signal transduction’ and ‘cell proliferation’. Two groups of genes involved in transcription from RNA polymerase II promoter were also upregulated. Among the downregulated genes, a large number belong to groups involved in DNA‐dependent transcription. The genes involved in anatomical structure morphogenesis and regulation of cellular processes and biological processes were also downregulated (Table 4). From the molecular function analysis, many GTPase genes were upregulated and the two largest groups of upregulated genes were involved in protein and DNA binding. Many different groups of genes involved in binding were downregulated along with groups of chemokine, cytokine and chemoattractant activity (Table 5).

After 3‐h starvation, a large number of transporters were found to be upregulated. For example, genes encoding transporters for carboxylic acid, organic acid, amino acid, and amine transport were upregulated. The largest group of upregulated genes belongs to the category ‘regulation of transcription, DNA‐dependent’ and also genes from the category ‘regulation of cell cycle’ were upregulated. One large group of downregulated genes were genes involved in metabolic processes. The genes from the categories ‘biosynthetic process’, ‘cell proliferation’, and ‘cell cycle’ were also downregulated (Table 4). Regarding molecular function, genes involved in transferase activity and ‘nucleic acid binding’ were downregulated. Genes involved in transmembrane transporter activity and transcription factor activity were upregulated (Table 5).

At 5 h, numerous genes involved in the category ‘metabolic process’ were found to be upregulated, together with genes coding for transporters involved in organic and carboxylic acid transport. Genes involved in sterol, cholesterol, and alcohol metabolism were all downregulated (Table 4). Upregulated gene categories belonging to molecular function were groups of genes involved in transmembrane transporter activity. Several genes involved in binding were also upregulated as well as genes involved in transcription and translation. Numerous binding genes were also downregulated, as well as genes involved in transferase, kinase and protein tyrosine/serine/threonine phosphatase activity (Table 5).

After 16 h of amino acid deprivation five different groups of metabolic processes were upregulated, ‘metabolic process’, nucleobase‐containing compound, RNA, tRNA, and cellular amino acid metabolic process. ‘RNA processing’, ‘RNA modification’, and ‘tRNA modification’ were also upregulated. Groups of genes involved in processes with lipids, cholesterol, and sterol were downregulated. The three largest downregulated groups of genes were ‘multicellular organismal development’, ‘organ morphogenesis’, and ‘anatomical structure morphogenesis’ (Table 4). From molecular function analysis, different groups of transmembrane transport activity were upregulated along with groups involved in transferase activity. The largest group of genes that were upregulated belonged to ‘nucleic acid binding’. Many groups of binding proteins were downregulated and also oxidoreductase, cytokine, and growth factor activity (Table 5).

### Heat map of the gene expression for SLCs and atypical SLCs

**Figure 3:**
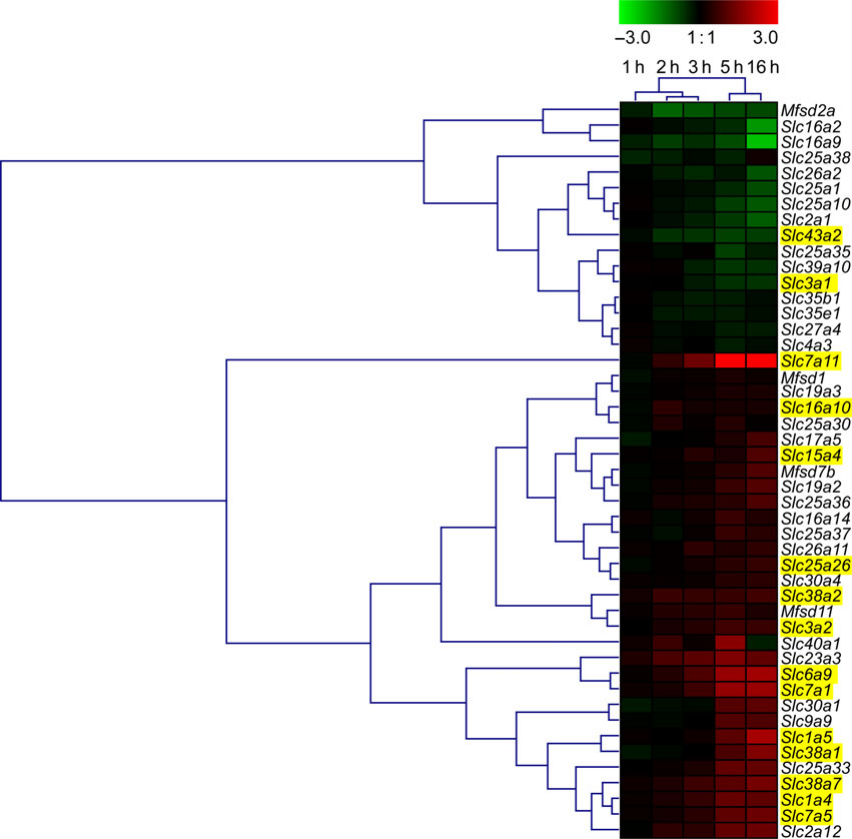
A heat map of the 47 regulated genes encoding SLCs or atypical SLCs. The heat map displays the alterations in gene expression for the starved cells compared with controls in the hypothalamic cell line N25/2 at 1, 2, 3, 5, or 16 h of amino acid starvation. The color scale represents the log2 difference for (1, 2, 3, and 16 h) and the log2fold change value (5 h) between starved and control cells. Green color represents downregulation and red color represents upregulation of gene expression. The genes and experiments were hierarchical clustered. Genes encoding amino acid transporters are highlighted in yellow.

A heat map over the alterations in gene expression (1–16 h) for all 47 genes encoding SLCs or atypical SLCs found to be significantly altered at 5 h of starvation was generated (Fig. 3). The genes and experiments were hierarchical clustered and the clustering displayed a clear time‐dependent effect with 1, 2, and 3 h clustering separately from the longer times, 5 h and 16 h.

### Verification of the microarray data using qPCR analysis

**Figure 4:**
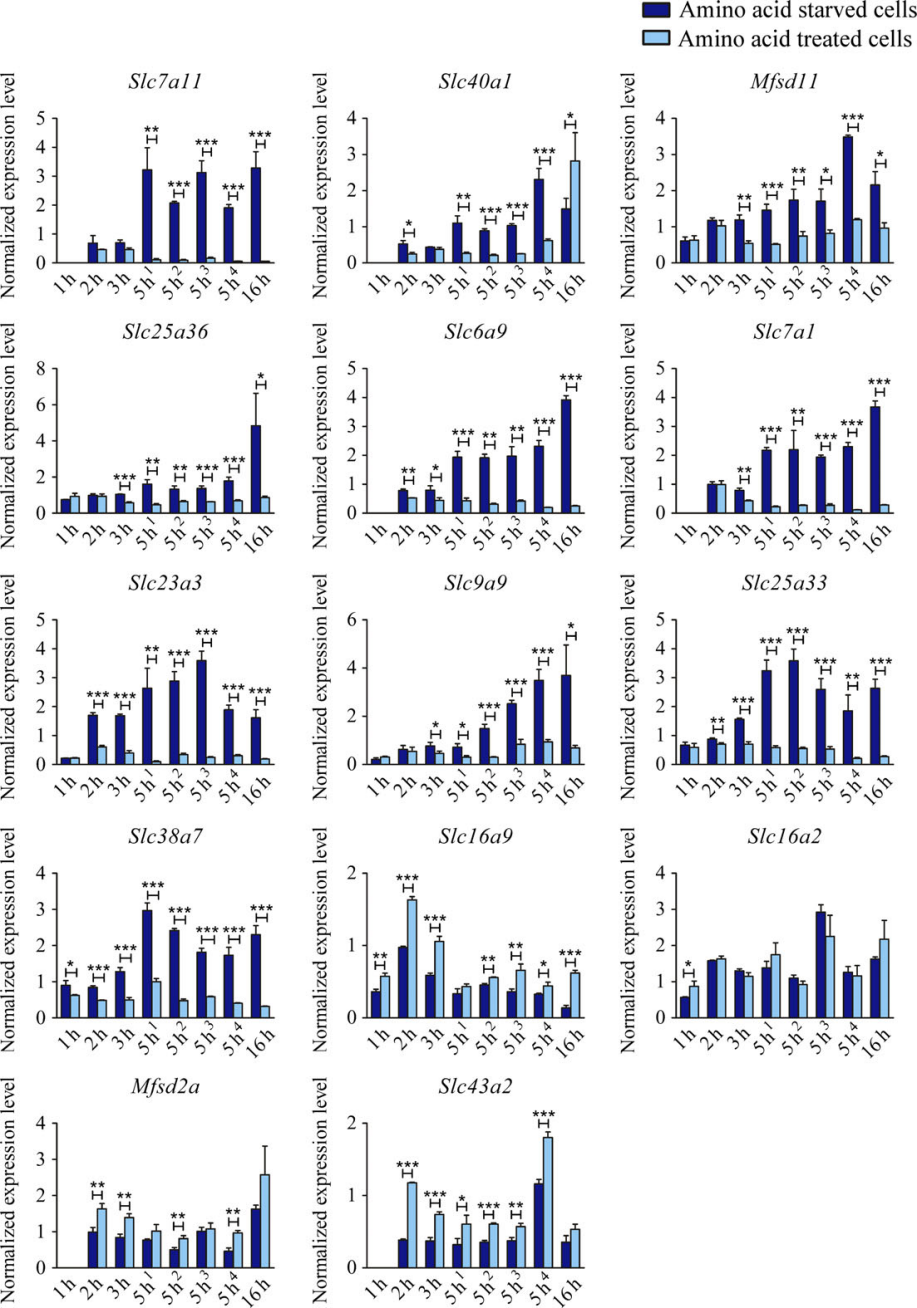
Gene expression data from the hypothalamic cell line N25/2 using qPCR to verify the microarray data. Normalized expression level ± SD,* n* = 3, (*n* = 4 for 16 h samples for *Slc16a2*,* Slc40a1*, and *Mfsd2a*). Unpaired *t*‐tests were performed, *≤0.05, **≤0.01, ***≤0.001 between starved cells and controls. Dark blue bars represent amino acid starved cells and light blue bars represent amino acid‐treated control cells. The *x*‐axis represents time of treatment in hours. Note that in some graphs, the data for 1 h of treatment are missing and the scale on *y*‐axis varies in the graphs.

The microarray data were verified using qPCR analysis for some of the genes found to be significantly altered at 5 h of starvation (Fig. 4). The genes analyzed were *Slc7a11*,* Slc40a1*,* Mfsd11*,* Slc25a36*,* Slc6a9*,* Slc7a1*,* Slc23a3*,* Slc9a9*,* Slc25a33*,* Slc38a7, Slc16a9*,* Slc16a2*,* Mfsd2a*, and *Slc43a2*. The results from the qPCR analysis comply with the results from the microarray, although the gene expression for *Slc16a2* was not significantly downregulated at 5 h of starvation using qPCR.

## Discussion

Complete amino acid starvation of the immortalized hypothalamic cell line N25/2 was performed and the gene expression alterations at 1, 2, 3, 5, or 16 h of starvation were analyzed using Affymetrix expression microarrays. At 5 h of starvation, expression levels of 1849 genes were significantly altered after adjustments for multiple testing; 1001 genes were upregulated, while 848 genes were downregulated. In this study, we decided to focus our analysis on genes encoding transporters from the SLC family, especially amino acid transporters and or putative amino acid transporters.

The 1849 genes identified for each incubation time were analyzed using GO annotations for biological process and molecular function to pinpoint where the gene clusters related to amino acid transport were regulated. The overall analysis showed that early, following 1–2 h of amino acid starvation, there were mainly changes in gene categories involved in basal cellular processes such as nucleic acid binding. GTPase‐related genes were also upregulated, whereas chemokine‐related genes and genes involved in metabolism were downregulated, suggesting that the cells respond with reduced growth. After 3 h and 5 h of starvation, genes involved in transport and metabolic processes were upregulated, possibly to increase intake and availability of amino acids and other substrates and genes involved in transferase activity and binding were downregulated. After 16 h of deprivation, there was upregulation of genes involved in RNA processes and transport activity and downregulation of genes involved in binding, lipid, sterol and cholesterol metabolism and organ morphogenesis, manifesting the assumption that the cells in general reacts with reduced growth, increased uptake and biomolecule synthesis. Taken together, genes involved in amino acid transport and amino acid transmembrane transporter activity were mainly upregulated at 3 h and 5 h, but amino acid transmembrane transporter activity was also upregulated following 16 h of amino acid starvation.

Forty‐seven genes encoding SLCs were found to be altered in the immortalized hypothalamic cell line at 5 h of starvation and these were divided into amino acid transporter encoding genes (15 genes) and genes encoding nonamino acid transporters (18 genes), orphan SLCs (10 genes), or atypical SLCs (4 genes). About 13 upregulated (*Slc7a11*,* Slc6a9*,* Slc7a1*,* Slc1a4*,* Slc7a5*,* Slc1a5*,* Slc38a7*,* Slc38a1*,* Slc3a2*,* Slc38a2*,* Slc25a26*,* Slc15a4*, and *Slc16a10*) and two downregulated (*Slc3a1* and *Slc43a2*) genes encoding amino acid transporters were found, see Table 2. Three members from the SLC7 family, the system y+ encoding gene *Slc7a1* (CAT‐1) 39, the system L encoding gene *Slc7a5* (LAT‐1) 40, and *Slc7a11* (xCT) from system xc‐ 41, had induced gene expression in the hypothalamic cell line, and all three were among the top five most upregulated amino acid transporters at 5 h of deprivation. *Slc7a1* 28, 42, *Slc7a5* 23, and *Slc7a11* 24 all have been found to respond with increased gene expression following amino acid starvation in several studies. *Slc7a11* forms a dimer with the heavy subunit *Slc3a2*, a cysteine/glutamate exchanger, belonging to system xc‐ 43. *Slc3a2* also heterodimerizes with *Slc7a5*, and forms a system L transporter for large neutral amino acids 43. *Slc3a2* has been found to be upregulated in human prostate cancer cells in an ATF4‐mediated way 26 and we also found induced gene expression of *Slc3a2* in the hypothalamic cell line. *Slc7a5/Slc3a2* together with *Slc1a5* are known to be involved in amino acid signaling and physiologically relevant activators in the mTORC1 pathway, where glutamine and leucine translocation is central, without this transport the activation is absent 44, 45. Moreover, the glycine transporter encoding gene, *Slc6a9* 46, was found to be the second most upregulated gene at 5 h of starvation in the hypothalamic cell line. Two members from the SLC1 family were found to be induced, the system ASC encoding genes, *Slc1a4* and *Slc1a5* 47. This is in concurrence with previous studies, where the expression of *Slc1a4* was upregulated in human HepG2/C3A cells following starvation 25 and *Slc1a5* was found to be upregulated in prostate cancer cells in an ATF4‐dependent way 26. Furthermore, three genes from the SLC38 family were upregulated. The expression of the system A encoding gene, *Slc38a2* was increased, which is in agreement with several studies in different cell types 21, 30, 38. More remarkably, there was also induced expression of *Slc38a1,* as well classified to system A 48. In a previous study, *Slc38a2* mRNA and protein expression were upregulated but no effect was seen on expression of *Slc38a1* 30. However, when SLC1A5 (*Slc38a5*) was silenced in cancer cells, SLC38A1 (*Slc38a1*) was found to be upregulated in an amino acid starvation response manner 49. Furthermore, in our study also the system N encoding gene *Slc38a7* was upregulated 50, which has not previously been shown to respond to amino acid levels.

Among the genes encoding nonamino acid transporters, orphan SLCs, or atypical SLCs, 32 genes (18 upregulated, 14 downregulated) were found, see Table 3. About 14 of the genes were orphans, including 4 atypical SLCs, and 18 genes encode transporters for e.g. sugars, ions, hormones, iron, fatty acids, and vitamins. Among the orphan SLC genes, the gene *Slc23a3* was the most upregulated one, and in the heat map (Fig. 3), it forms a cluster with the glycine transporter *Slc6a9* (GLYT1) 51 and the cationic l‐amino acid transporter *Slc7a1* 43, indicating that they are regulated in a similar way. The SLC23 family, the Na^+^‐dependent ascorbic acid transporter family, with four members, have so far two characterized l‐ascorbic transporters 52. *Slc23a3* belongs to the Pfam clan Amino acid‐Polymine‐organoCation (APC)–superfamily. The APC clan also encloses the amino acid transporter‐encoding genes, *Slc7a11* 24, *Slc7a1* 42, *Slc7a5* 23, and *Slc38a2* 27, known to be upregulated in response to amino acid starvation. It is therefore possible that *Slc23a3* also could encode a transporter with preference for amino acids. In addition, we found 13 genes belonging to the major facilitator superfamily (MFS) clan, encoding putative SLC transporters, *Mfsd1*,* Mfsd2a, Mfsd7b*, and *Mfsd11* or genes encoding SLC transporters *Slc2a1*,* Slc2a12*,* Slc16a2*,* Slc16a9, Slc16a14*,* Slc17a5*,* Slc19a2*,* Slc19a3*, and *Slc40a1*. The MFS family is the largest group of phylogenetically related genes with the SLC superfamily, and at least 13 of the SLC families belong to the MFS clan 53. *Mfsd11* was upregulated and had a similar regulation pattern in the heat map as the amino acid transporter genes, *Slc3a2* and *Slc38a2*. Furthermore, the orphan gene *Mfsd2a* were among the most downregulated genes at 5 h of starvation, while the two orphan members *Mfsd7b* and *Mfsd1* were upregulated. We found four members, which are phylogenetically closely related 54, from the SLC16 family. *Slc16a2* and *Slc16a9* were downregulated while *Slc16a10* and *Slc16a14* were upregulated in the hypothalamic cell line. *Slc16a9* and *Slc16a14* are orphans, while *Slc16a2* (MCT8) is a transporter for thyroid hormones 55, and *Slc16a10* (MCT10) is a transporter for thyroid hormones 56 and the aromatic amino acids tryptophan, tyrosine, and phenylalanine, classified to system T 57, 58. It is possibly that *Slc16a14* could encode a transporter for amino acids.

The immortalized cell line N25/2 used in this study is in many aspects different from normal neuronal cells. For example, N25/2 cells divide readily while primary neuronal cells do not. Still, the N25/2 cells have retained many neuronal characteristics such as formation of synapse like structures and expression of neuronal markers such as neuro N and synaptic vesicle proteins 2 (SV2). The cell line was also originally created by infection of mouse embryonic hypothalamic cultures with SV40 retroviruses (https://www.cedarlanelabs.com/Products/Detail/CLU110?lob=AllProducts). This would likely result in fewer genetic changes than what is found in a cell line derived from tumors. However, the *in vivo* validity of the present results needs to be further investigated, preferably in whole animals or in primary neuronal cells. In our large‐scale study, we have screened the entire mouse genome for genes responding to amino acid deficiency, and we have not measured any changes on protein level. However, the alterations in gene expression found for several SLC genes needs to be further investigated on protein level, to better reflect cellular function.

The fact that we found several genes, *Slc7a1*,* Slc7a5*,* Slc7a11*,* Slc3a2*,* Slc1a4*,* Slc1a5*, and *Slc38a2*, upregulated in our study, as previously shown to be induced by amino acid starvation in several cell lines, reinforces the validity of our microarray data. Moreover, we also found increased gene expression for the amino acid transporter encoding genes *Slc6a9*,* Slc38a1*,* Slc38a7*,* Slc25a26*,* Slc15a4*, and *Slc16a10*, not previously known to respond to altered amino acid levels. In addition, we also found genes encoding orphan SLCs, e.g., *Slc23a3* and *Slc16a14* among others, which possibly encode transporters with preference for amino acids. Our data therefore suggest that numerous of the genes found to be regulated in this study could be involved in amino acid sensing and signaling pathways and could hold responsive elements.

## Conclusions

In this study, 1001 genes were significantly upregulated and 848 genes were significantly downregulated of 28 270 genes in the immortalized mouse hypothalamic cell line N25/2, at 5 h of amino acid starvation. Among these 1849 genes, 47 genes were SLCs or atypical SLCs. About 15 genes encoding SLC amino acid transporters were found, *Slc7a11*,* Slc6a9*,* Slc7a1*,* Slc1a4*,* Slc7a5*,* Slc1a5*,* Slc38a7*,* Slc38a1*,* Slc3a2*,* Slc38a2*,* Slc25a26*,* Slc15a4*, and *Slc16a10* were upregulated while only two genes, *Slc3a1* and *Slc43a2*, were downregulated. At 5‐h deprivation, genes encoding amino acid transporters from system A, ASC, L, N, T, xc‐, and y+ were upregulated. We also found, according to GO annotations, that the gene clusters involved in amino acid transport and amino acid transporter activity were most upregulated at 3 h and 5 h of amino acid starvation.

## Author contributions

SH wrote manuscript, designed study, performed starvation experiment, analyzed data, and performed statistical analysis. EL performed qPCRs, analyzed qPCRs, and wrote part of manuscript. TA performed qPCRs and analyzed qPCRs. RF wrote manuscript, designed study, analyzed data, and performed bioinformatic analysis.
